# AI and the PI: Infinite Generation, Finite Understanding

**DOI:** 10.1021/acs.jcim.6c03139

**Published:** 2026-10-06

**Authors:** Toni Giorgino

**Affiliations:** † Istituto di Biofisica, 9327 Consiglio Nazionale delle Ricerche (CNR-IBF), via Celoria 26, I-20133 Milan, Italy

## Abstract

Generative artificial intelligence (AI) produces code and prose quickly, apparently enabling a principal investigator (PI) in a computational group to do more. Assessing this gain must also account for the laboratory’s role in training scientists who understand, maintain, and own their work. AI creates a generation–verification asymmetry: it reduces the effort of producing plausible code and prose without similarly reducing the expertise needed to evaluate them, potentially leaving groups with technical and epistemic debt while obscuring the reasoning needed for supervision. I argue that AI adoption should therefore be judged by whether it builds sustainable laboratory capacity, with assistance adjusted to task purpose and verifiability and human ownership retained for every consequential artifact.

## Graphical abstract

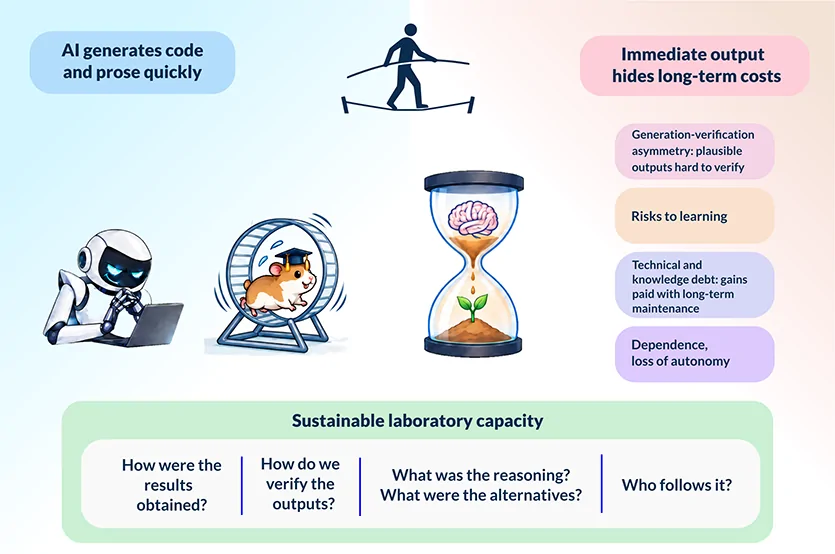

## Introduction

Since generative large language models (LLMs) entered everyday laboratory work, an overloaded principal investigator (PI) can ask a model to fill the umpteenth bureaucratic form, draft analysis code, summarize an unfamiliar method, or outline a paper. Trainees have access to the same capabilities. Work appears soon, prose is smooth, and an empty source file becomes a program in minutes. The bargain is tempting because its profit is visible; the pound of flesh it exacts is harder to measure.

Recent discussions have considered how PIs in computational chemistry can promote critical thinking, effective mentorship, and responsible use of rapidly developing computational tools.^1,2^ In this Viewpoint, I focus more narrowly on how generative AI changes the relation between scientific output, understanding, and supervision in small, compute-intensive research groups, drawing on my own, necessarily partial experience in computational biophysics; groups differ substantially in size and organization, so the opinions below are just provisional views. Questions of scientific authorship, biases, and the broader, even existential, risks of AI remain outside my scope.^3,4^

Uncritical generative-AI use can produce a laboratory-scale analogue of disempowerment, eroding human influence over scientific work when it replaces the cognition and labor through which expertise develops.^5^ For PIs in data-, model- and code-intensive research, the question is therefore how routine AI use changes the group’s long-term capacity.

## A Laboratory Produces Results and Researchers

A research project has two outputs: tangible artifacts (a table, program, or paper); and a researcher with greater scientific judgment and practical command of the work. These outputs can now diverge: researchers may produce similar papers while acquiring very different capacities.^6^

When a trainee presents a result, I wish to understand how they reached it. Given time and luck, I could perhaps reach the same end point myself; but I long to know why they chose an approach, where it failed, how confident they are, and especially which promising alternatives emerged.

A figure’s author usually knows more than the image shows. AI-mediated workflows conceal this information behind polished output, and retrospective explanations cannot fully recover the decisions (or lack thereof) that produced it. Reporting only the end point deprives the PI of information needed to evaluate the science and guide the scientist.

Computational scientists and engineers already appreciate *technical provenance*: a record of materials and procedures reproducibly linking an input to a claim.^7,8^ *Epistemic provenance* adds the human dimension: who understands the process and “owns” consequential choices, which alternatives were rejected and why, and where uncertainty remains.

## AI, a Dark Mirror

Generative AI reflects the ability brought to it, enhancing or distorting it. Producing a candidate answer has become dramatically cheaper; establishing its scientific and architectural suitability has not. An expert anticipates intended behavior and recognizes assumptions; a novice may receive code more sophisticated than they could independently create or repair.

This way, LLMs create an asymmetry in the *generation–verification burden*. Overreliance on generated code can particularly harm junior researchers: they have less expertise to identify unsuitable output, while the work AI displaces would help them develop it. (Incidentally, the same asymmetry could spell the doom of the traditional peer-review model.)

*Automation bias* (the tendency to rely on an automated recommendation) exacerbates the problem by encouraging reliance beyond the system’s actual capabilities. Repeated deference reduces scrutiny and may gradually erode the judgment needed to recognize unsuitable outputs.^9,10^ Broader treatments examined overreliance and cognitive offloading^11^ and their effects on human thought.^4,12^

An useful question is to which extent users can still independently and meaningfully verify AI output. Extensive automation becomes less attractive as verification becomes harder, and the scientific consequences of error increase.

## Learning after AI-Assisted Practice

Education research has long distinguished successful performance during practice from durable learning which transfers to a new situation. Generative AI makes this distinction crucial because cognitive offloading can improve apparent performance without consolidating knowledge and judgment.^13,14^

Explaining an idea often reveals gaps in understanding, and helps people reorganize what they know. When AI converts unformed thoughts directly into polished prose, it removes an act that would have revealed the gap.^15^

Struggle is part of computational science, too. Tracing an inconsistency or odd data point may reveal an unexpected feature of the data or method (“that’s funny”), and trying out alternatives clarifies which choices matter. Routine inconvenience deserves automation, but researchers also need direct encounters with uncertainty, and persistence, in order to build a mental model of the problem.

The border between useful struggle and automation is partly pedagogical. LLMs editing an already formed argument for clarity presents relatively little risk; asking a model to develop it or supply apparent novelty can let weak arguments pass unnoticed. Similarly, generating routine code for a familiar method differs from delegating an unfamiliar analysis the researcher was expected to learn. The PI should make the call according to the task’s purpose.^16^

## Scientific Code and Debt

Code and data are central to computational laboratories: algorithms materialize methods and become part of the scientific discourse.^17^ Well before generative AI, guidance on scientific computing emphasized version control, readable and documented code, automated testing, and reproducible workflows.^7,18–20^ The FAIR principles likewise call for research software to remain usable by others.^21^ For molecular dynamics, for example, JCIM’s reporting guidelines set requirements for methodological detail, convergence assessment, data availability, and comparison with experimental evidence where available.^22^

These practices are meant to limit *technical debt*, i.e. the future maintenance burden created by expedient choices. The “reproducibility bill” often hits only after a trainee leaves, or a reviewer requests another analysis. The PI or a new student then spends the supposedly saved time validating code and reconstructing its intent. Unowned code is a liability even when cheap to produce.^23^

AI can also streamline testing, documentation updates, refactoring, adherence to coding conventions, and software chores in general. LLM-assisted refactors to AMBER’s LEaP program, for example, accelerated it and expanded the treatable system sizes.^24^ However, good scientific code depends on context and expected change: an explicit transformation may be preferable to a general abstraction to make an assumption visible; a minimal readable script may suit a short-lived purpose, whereas a reusable pipeline may need a stable interface for foreseeable applications.

Rapidly generated scientific software still needs someone to keep it interpretable and maintainable. When code generation outpaces understanding and review, technical debt becomes *a knowledge debt*: code or results one cannot fully understand or defend.^25^ A program may run and pass its tests even when nobody can explain why its architecture suits the scientific question, which assumptions affect the result, or how to adapt it as the project changes. Running code and passing tests do not guarantee scientific validity.^9^ Plausible outputs may easily conceal inappropriate populations, invalid comparisons, data leakage, or unsuitable defaults or numerical methods. AI-assisted research requires reproducibility, uncertainty assessment, and explanation at least as much as ever.^2^

## Middleman Trap

A member who mostly relays a PI’s question to a tool makes their contribution difficult to defend. I need the trainee’s background, fresher coursework memories than mine, developing judgment, and eventually their independent scientific direction. Their contribution lies in framing the problem, recognizing inconsistencies, examining literature I may have read cursorily, and suggesting which alternatives deserve attention.

As execution becomes cheaper, question selection, judgment, interpretation, and responsibility become relatively more valuable. Yet researchers develop ideas and scientific taste partly through execution: discovering which apparently simple tasks are not simple, which controls provide more information, and which attractive directions fail. The execution AI displaces is therefore also part of the apprenticeship for original work.

PIs are not immune to similar risks: habitual delegation can reduce their contact with methods, code, and data. Confidence becomes tied to tool availability, while the ability to detect failures weakens. This re-enacts a classical dilemma of automation: humans remain responsible for exceptional cases while losing the routine practice that built the skills to handle them.^26^

## Practical Principles for AI-Assisted Laboratory Work

Blanket bans are impractical to enforce and deny researchers experience with increasingly ubiquitous tools. Laboratories nevertheless need explicit practices for AI-assisted work. The arguments above suggest five provisional principles.

1. **State the immediate objective.** Appropriate assistance depends on whether a task primarily serves production or learning. Substantial automation is reasonable for an urgent result using a well-understood method. Tasks intended partly to develop expertise warrant greater direct involvement.
2. **Calibrate AI use on verifiability and scientific stakes.** Extensive automation suits tasks whose correctness is cheap to establish, such as formatting or mechanical refactoring. Involve more direct human expertise when errors are difficult to detect or affect scientific interpretation.
3. **Love the result and the path.** Reports should identify important assumptions, rejected approaches, remaining uncertainty, and substantial AI contributions. Full transcripts are too verbose and rarely provide this information. The record should support reconstructing the analysis and reveal the researcher’s understanding.
4. **Stress human ownership.** Someone in the laboratory must be able to explain, test, modify, and justify every consequential artifact. “The AI produced it” provides neither provenance nor an excuse. Review and revision are statements of ownership.
5. **Assess confidence independently.** An LLM’s confident wording provides no useful evidence and may distort a researcher’s own assessment. Appropriate controls, explicit checks, and independent reasoning should determine confidence in generated work.

These principles tie AI use to the task, the user’s expertise, verification difficulty, and the scientific consequences of error, rather than prescribing a “fixed amount”. Supporting Information, Text S1 proposes a tentative translation of these principles into a laboratory policy.

## Conclusions

Generative AI creates a recurrent supervisory problem in computational research: an LLM-assisted plot or substantial program may arrive quickly and look polished, yet come with little explanation of why the analysis answers the research question. The laboratory gains an artifact sooner; whether it gains capacity is less clear.

AI reshapes supervision into management of a human–machine system. The PI remains accountable for papers and software, but the path from question to result becomes harder to inspect. Raising output targets can thus leave the PI with more artifacts to validate and less information to assess them. AI adoption should therefore be judged by *sustainable*, *understandable*, and *accountable scientific capacity rather than output alone*. Groups must decide where automation helps while preserving scientific direction, human development, and ownership.

These viewpoints require empirical evaluation and will need to be revisited, especially as agents capable of autonomous research workflows emerge.^27–31^ The need to preserve human understanding is unlikely to disappear. If anything, as AI assumes a greater share of execution, cultivating understanding may become even more central to scientific disciplines themselves.^32^

## Supplementary Material



## Notes

A tentative policy: practical guidance for generative AI use (PDF)
