# Supplementary material for: AI and the PI: Infinite Generation, Finite Understanding
Source: J Chem Inf Model. 2026 Oct 6;66(20):13019–22. doi: 10.1021/acs.jcim.6c03139 (PMC13715480; doi:10.1021/acs.jcim.6c03139)
Supplement: Supplementary file 1 [file ci6c03139_si_001.pdf]

Supporting Information

AI and the PI: Infinite Generation, Finite Understanding

Toni Giorgino

A tentative policy: guidance for generative AI use.

This illustrative policy translates the article's principles into practical guidance. It is provisional, and will require adaptations to the specific contexts, groups and types of research. It has not yet been evaluated quantitatively as an intervention.

> “
>
> I am not against the use of AI assistants, but you must understand what you are doing and its consequences. They can generate plausible-but-wrong information, plausible-but-empty prose, and superficially correct code with subtle defects. All are serioud: errors can mislead, and empty prose wastes the reader's attention. Experts may "feel" something is off even without being certain of its cause, and be annoyed. Non-native authors are especially exposed in this regard (erroneously believing AI prose is natural).
>
> In summary, if you haven't understood the code or the problem, you will not be able to establish whether the AI's output is sound. LLMs will nevertheless put out pages and pages of confused code and text for you to figure out, which will be practically impossible to validate.
>
> As a rule of thumb, the utility of a code assistant increases with your expertise and ability to verify its output. The appropriate balance also depends on whether a task is intended primarily to produce a result or to help you learn. If you are a junior researcher, especially a thesis student, your tasks may have been structured for their training value. AI-driven detours may appear to produce answers while denying you that training, slowing the real progress of the thesis, and creating additional code and text that must be checked and justified. Learning to navigate AI assistants will require experimentation. Remember that the value of many scientific tasks, including a thesis, lies both in their outputs and in the process, difficulties, and understanding encountered while completing them.

In general, try to have LLMs do work "for you", not "instead of you". The distinction may be blurred and doing this right will need experimentation and balance on your part.

Do not upload unpublished results, personal data, controlled genomic data, confidential manuscripts, or other sensitive material to an external system unless its use has been approved.

Hints when writing

1. **Disclose your use.** Tell your supervisor when and how you have used an AI assistant. There is no need to hide it. Disclosure makes it possible to review the work appropriately and to discuss whether the tool helped or hindered your work. When its contribution is substantial, keep a short record of what it contributed and of the important assumptions or choices you checked.

2. **Do not delegate your ideas or creativity.** Own the formation of your scientific ideas and the outlines of your arguments. An LLM may suggest alternatives for you to criticize; however generated outlines are likely to sound formulaic; evaluating apparently-new ideas may be harder than producing your own. Do not expect the tool to supply the creativity that will distinguish your work. And yet, creativity is one of the things that will make you stand out in the future. Exercise it.

3. **A reasonable use is to have them check your text**, for clarity, for example, or to reword it after you have formed the argument yourself. You remain responsible for checking the meaning, factual claims, and references in the final text.

4. **Check originality and venue requirements.** Treat generated prose as material of uncertain provenance. Check source overlap, attribute borrowed ideas and wording, and follow the disclosure policy of the intended journal or institution. Writing assistants raise the chance that you could be accused of being too close to someone else's words, even in the distant future. [1]

Hints when coding

You may try to use LLMs for coding, maybe in tools such as VS Code or Codex, but beware: it is not as obvious as it may seem. For genuinely new tasks, it may create superficially correct code with subtle defects in logic. In my experience, overusing AI-generated code is likely to produce more harm than benefit, especially for junior researchers. You may be trading short-term apparent benefit for mid-term harm.

[1] `https://www.carlsonlab.bio/thoughts/the-only-reason-youll-ever-need-not-to-write-with-ai`

1. **Make your own preliminary attempt.** Before asking an AI to write code or solve an analysis problem, first consider whether the task is intended primarily for production or learning. If it is meant to help you learn, or if the method is unfamiliar, try to formulate the problem and make your own preliminary attempt. Otherwise, you may never be able to judge whether the generated answer addresses the actual task.

2. **You are expected to know how and why the code works or why it does not.** It is ultimately your responsibility. You must be able to explain, test, modify, and justify anything you submit or use.

3. **"The AI made it" is not an argument.** Seems obvious, but the decision to use its output remains yours. Dropping ownership is a recipe for failure and/or for being replaced by an AI.

4. **Use AI mainly when correctness is easy to verify.** AI-created bugs can be subtle even when the code looks superficially correct, and may increase debugging time many-fold. It is most sensible to use generated code for tasks whose correctness can be established immediately and independently. Do not take confident wording as evidence: devise appropriate tests and independent checks.

5. **Check architecture, logic, and documentation.** Some AI-created "bugs" are actually deep architectural misunderstandings or logical flaws. A generated program may appear to run nevertheless. The same warning applies to generated code documentation: a plausible explanation is not proof that either the explanation or the code is correct.

6. **"Code is a liability, not an asset".** Code exists to solve a problem; if it does not solve one, it creates one. Once the purpose has been achieved, less code and less complexity are usually better. Do not accept pages of generated code that you cannot understand or maintain.
